# Human umbilical cord mesenchymal stem cells derived extracellular vesicles regulate acquired immune response of lupus mouse in vitro

**DOI:** 10.1038/s41598-022-17331-8

**Published:** 2022-07-30

**Authors:** Min Xie, Cuifang Li, Zhou She, Feifeng Wu, Jueyi Mao, Marady Hun, Senlin Luo, Wuqing Wan, Jidong Tian, Chuan Wen

**Affiliations:** Division of Hematology and Tumor, Children’s Medical Center, The Second Xiangya Hospital, Central South University, Changsha, Hunan People’s Republic of China

**Keywords:** Immunology, Adaptive immunity, Applied immunology, Autoimmunity, Immunotherapy, Transplant immunology

## Abstract

Systemic lupus erythematosus (SLE) is an autoimmune disease involving multiple systems. Immunopathology believes that abnormal T cell function and excessive production of autoantibodies by B cells are involved in multi-organ damage. Human umbilical cord mesenchymal stem cells (hUCMSCs) therapies have endowed with promise in SLE, while the function of MSC-derived extracellular vesicles (MSC-EVs) was still unclear. Extracellular vesicles (EVs) are subcellular components secreted by a paracellular mechanism and are essentially a group of nanoparticles. EVs play a vital role in cell-to-cell communication by acting as biological transporters. New evidence has shown beneficial effects of MSC-EVs on autoimmune diseases, such as their immunomodulatory properties. In this study, we investigated whether hUCMSCs derived extracellular vesicles (hUCMSC-EVs) could regulate abnormal immune responses of T cells or B cells in SLE. We isolated splenic mononuclear cells from MRL/lpr mice, a classical animal model of SLE. PBS (Phosphate-buffered saline), 2 × 10^5^ hUCMSCs, 25 µg/ml hUCMSC-EVs, 50 µg/ml hUCMSC-EVs were co-cultured with 2 × 10^6^ activated splenic mononuclear cells for 3 days in vitro, respectively. The proportions of CD4^+^ T cell subsets, B cells and the concentrations of cytokines were detected. Both hUCMSCs and hUCMSC-EVs inhibited CD4^+^ T cells, increased the production of T helper (Th)17 cells, promoted the production of interleukin (IL)-17 and transforming growth factor beta1 (TGF-β1) (*P* < 0.05), although they had no significant effects on Th1, Th2, T follicular helper (Tfh), regulatory T (Treg) cells and IL-10 (*P* > 0.05); only hUCMSCs inhibited CD19^+^ B cells, promoted the production of interferon-gamma (IFN-γ) and IL-4 (*P* < 0.05). hUCMSCs exert immunoregulatory effects on SLE at least partially through hUCMSC-EVs in vitro, therefore, hUCMSC-EVs play novel and potential regulator roles in SLE.

## Introduction

In recent years, animal experiments and clinical evidence have demonstrated that stem cell transplantation could treat refractory systemic lupus erythematosus (SLE) and improve its prognosis. Therefore, stem cell transplantation has become an important treatment method for SLE and has been widely used in clinical practice^1,2^. At present, adult stem cells for treating refractory SLE are mainly derived from hematopoietic stem cells (HSCs) and mesenchymal stem cells (MSCs)^3–5^. Allogeneic HSCs transplantation could result in a high incidence of graft-versus-host disease (GVHD) and high transplant-related mortality. While autologous HSCs transplantation does not fundamentally correct the abnormal immune system of SLE, leading to a high recurrence rate after transplantation^4,6^. These problems have been troubling the clinical application of HSCs transplantation in SLE, and have become the technical bottleneck of stem cell transplantation. Fortunately, studies indicated that MSCs have brought us a new opportunity in recent years.

Due to its features of low cellular immunogenicity and strong immunomodulatory function, MSCs have brought great hope for the treatment of autoimmune diseases, especially SLE. There are studies suggested that MSCs transplantation can reduce the production of inflammatory factors and autoantibodies, and promote the repair of lupus nephritis (LN)^7^. However, the detailed mechanisms underlying the treatment of LN by MSCs transplantation remain unclear. Current studies have also shown that MSCs may replace damaged renal parenchymal cells by differentiating into functional cells, which is called the differentiation mechanism. However, some scholars believe that MSCs cannot regenerate renal parenchymal organs by differentiating into plenty of renal parenchymal cells^8^. In addition, other researchers believe that MSCs play an immune effect to improve kidney damage by secreting anti-inflammatory biological factors (such as IL-10, PGE2, IL-1R, TGF-β1), which is the secretion mechanism^9^. For example, scholars only use the supernatant obtained from MSCs to treat diseases, and the survival of renal tubular cells was also independently promoted^10^. These findings suggest that MSCs may reshape immune function through the secretion mechanism to treat LN. However, the detailed secretion mechanism of MSCs remains unclear.

Extracellular vesicles (EVs) are nanoscale membrane vesicles actively released by cells and have a diameter ranging between 50 and 2000 nm with a bilayer lipid membrane^11^. According to the diameter of membrane vesicles, EVs are classified into exosomes, microvesicles, and apoptotic bodies, currently the former two are the most widely studied. EVs enrich with proteins (usually the tetraspanins CD9, CD63 and/or CD81) or other molecules^11,12^. These active cargos are transferred to recipient cells through EVs to mediate the communication between parent cells and recipient cells, and thus participating in physiological and pathological processes such as immune response, cell phenotype regulation, and angiogenesis^13^. Recent studies have shown that mesenchymal stem cells derived extracellular vesicles (MSC-EVs) play an important role in autoimmune diseases. In addition, the transfer of signal molecules to immune cells through MSC-EVs mediated by MSCs has become a novel mechanism for the immunotherapeutic effect of MSCs^7^. Researches have indicated that MSCs-EVs have therapeutic effects in a variety of disease, such as GVHD^14^, type 1 diabetes^15^ and inflammatory arthritis^16^. In 2019, the finding indicated that MSC-EVs inhibit inflammatory responses by regulating immune cells in inflammatory tissues^17^. The polarization and plasticity of macrophages may play an important role in the pathogenesis and progression of SLE^18^. Research has reported the regulatory effects of MSC-EVs on macrophage polarization. For example, MSC-EVs promoted polarization of M1/M1-like macrophages to a M2/M2-like state in vitro^19–22^. Infusion of MSC-EVs switch macrophage phenotype to M2 polarization in colon tissue and prevented disease progression in a mouse model of colitis^20,23^ and increased M2 macrophages in the spleen or lung in the MRL/lpr mouse model of SLE^24,25^. However, the effects of MSC-EVs on acquired immune response in SLE remain unclear in related researches. Therefore, in this study, we investigated whether hUCMSC derived extracellular vesicles (hUCMSC-EVs) could regulate abnormal immune responses of T cells or B cells in SLE.

## Results

### Characterization of hUCMSC-EVs

**Figure 1:**
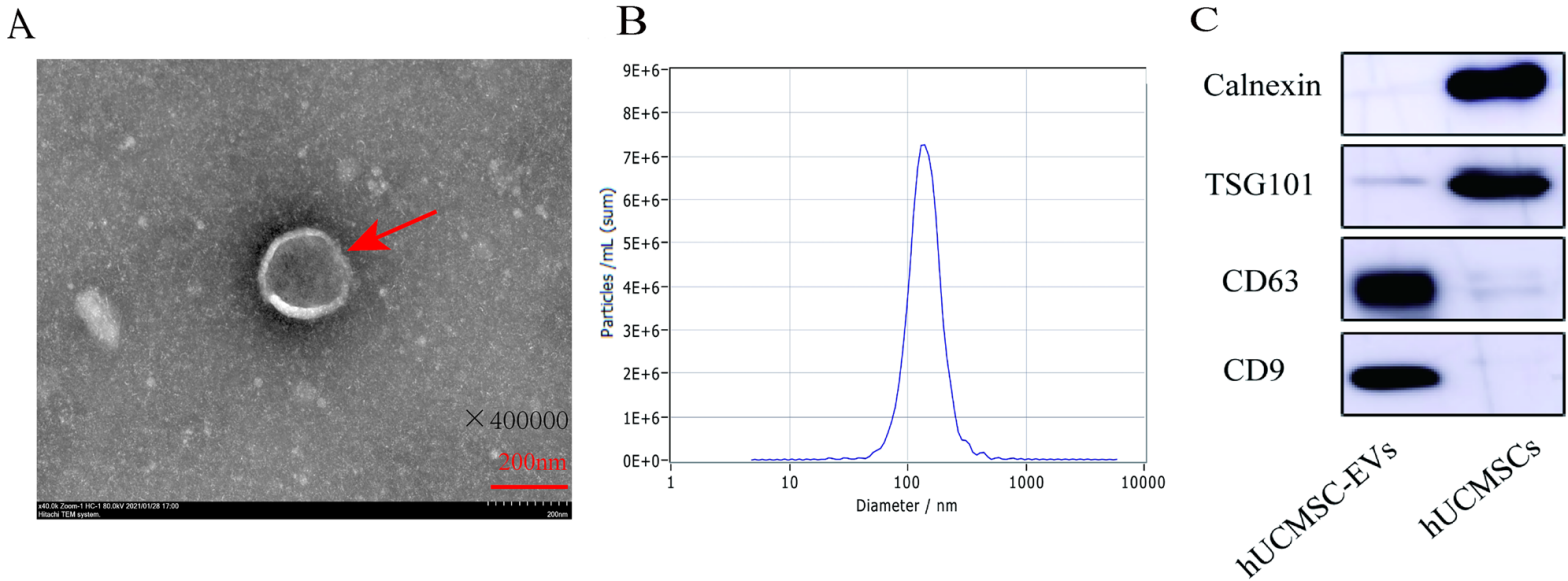
hUCMSC-EVs characterization. (**A**) Transmission electron microscopy (TEM). (**B**) NTA particles size distribution. (**C**) Western blot.

hUCMSCs were cultured in the MSCs serum-free complete medium until cells reached 80% confluency and then switched to MSCs basic medium that was free of protein and avoiding contamination of exogenous protein or EVs. We then separated hUCMSC-EVs from harvested supernatants by differential ultracentrifugation. The isolated fraction was assessed by transmission electron microscopy (TEM), Nanoparticle tracking analysis (NTA), and Western blotting to ensure high-quality sample was obtained. hUCMSC-EVs were showed a bilayer-membrane structure of about 150 nm in diameter at TEM analysis (Fig. 1A). Subsequently, NTA analysis indicated that the extracellular vesicles were approximately 139.1 nm in diameter (Fig. 1B). As expected, when compared with the cell lysate counterparts, hUCMSC-EVs were enriched in conventional exosome-associated biomarkers (e.g. CD9, CD63 and TSG101), while calnexin, a negative marker of small EVs, was not detected (Fig. 1C). These results indicated that hUCMSC-EVs were successfully isolated.

## Regulation of hUCMSC-EVs on T and B cells in MRL/lpr mice splenic mononuclear cells

### hUCMSC-EVs inhibited CD4^+^ T cells in MRL/lpr mice splenic mononuclear cells in vitro

**Figure 2:**
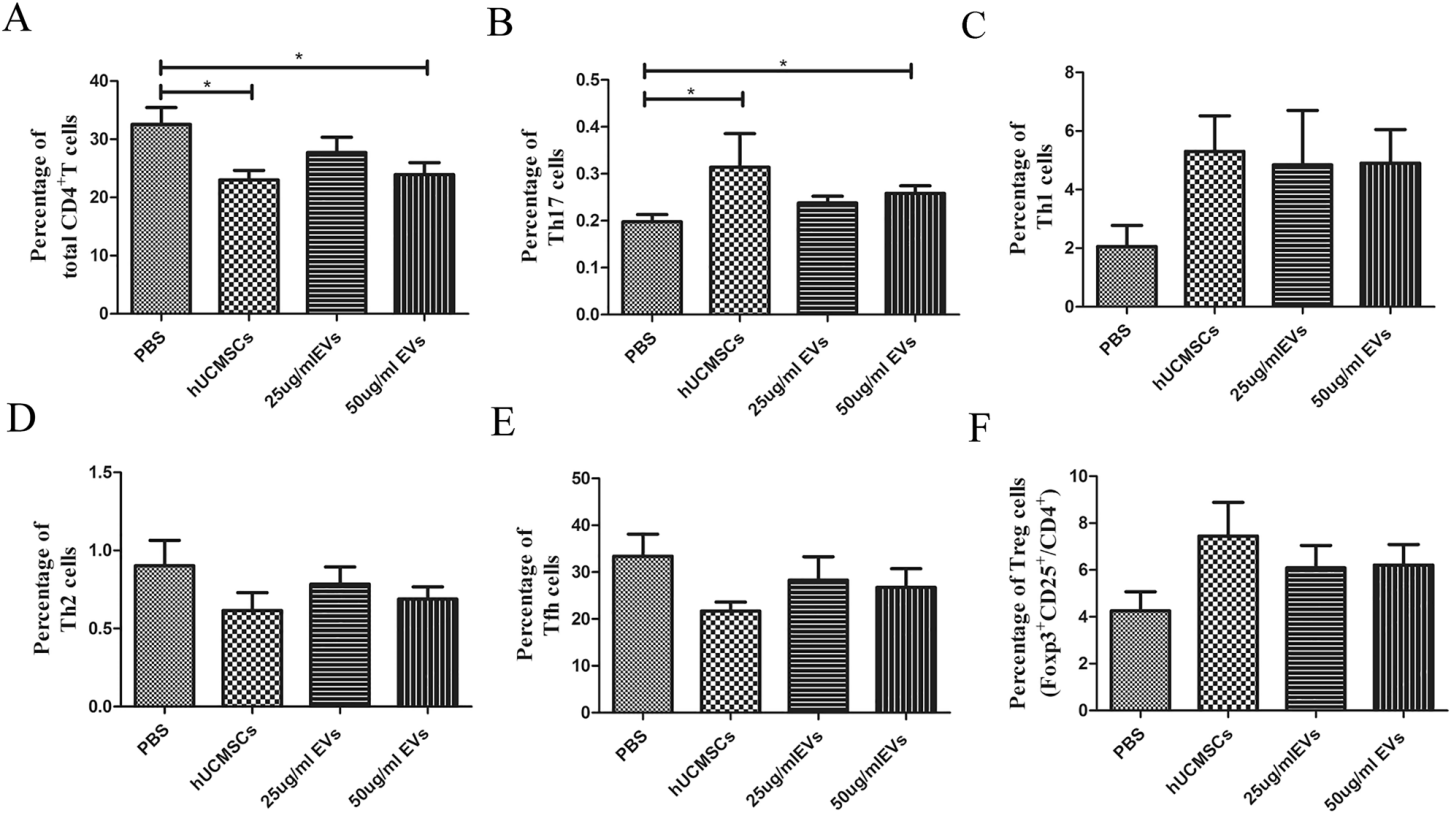
Immunomodulatory effects of hUCMSCs and hUCMSC-EVs on MRL/lpr mouse splenic T cells in vitro. Flow cytometric analysis of the proportion of CD4^+^ T cell (**A**), CD4^+^ T cell subsets [Th1 (**B**), Th2 (**C**), Th17 (**D**), Tfh (**E**), Treg (**F**)] in splenic mononuclear cells. Data were expressed as the means ± SEM. Data were analysed by one-way ANOVA with Tukey's post hoc test or Kruskal–Wallis test with Dunn's post hoc test, *P < 0.05.

Different concentration of hUCMSC-EVs was cocultured with splenic mononuclear cells. After undergoing for a three-day cocultured with hUCMSC-EVs, CD4^+^ T cells was inhibited. The results showed that the proportion of CD4^+^ T cells in hUCMSCs group (23.03 ± 1.635%) or 50 μg/ml hUCMSC-EVs (23.93 ± 2.042%) group was lower than that in PBS control group (32.56 ± 2.880%), the difference was statistically significant (*P* < 0.05, Fig. 2A, Fig. S1). The proportion of CD4^+^ T cells in hUCMSCs group was lower than that in 50 μg/ml hUCMSC-EVs group, while there was no significant difference (*P* > 0.05, Table S1). It indicates that the effects of 50 μg/ml hUCMSC-EVs in the inhibition of CD4^+^ T cells were comparable with 2 × 10^5^ hUCMSCs.

### hUCMSC-EVs promoted Th17 cells differentiation in MRL/lpr mice splenic mononuclear cells in vitro

To investigate the effect of hUCMSC-EVs and hUCMSCs on Th17 cell differentiation, activated mouse splenic mononuclear cells by anti-CD3e and anti-CD28 were cocultured with hUCMSC-EVs or hUCMSCs for 3 days. Both hUCMSC-EVs and hUCMSCs promoted Th17 cells differentiation. Flow cytometric analysis showed that the proportion of Th17 cells in hUCMSCs group (0.363 ± 0.007%) or 50 μg/ml hUCMSC-EVs group (0.258 ± 0.017%) was higher than that in PBS control group (0.198 ± 0.015%), the difference was statistically significant (*P* < 0.05, Fig. 2B, Fig. S2). The effects of 50 μg/ml hUCMSC-EVs in the differentiation of Th17 cells were comparable with 2 × 10^5^ hUCMSCs (*P* > 0.05, Table S2).

### hUCMSC-EVs had no significant effects on other CD4^+^ T cell subsets in MRL/lpr mice splenic mononuclear cells in vitro

Addition of hUCMSC-EVs to splenic mononuclear cells did not affect the proportion of the Th1 (2.055 ± 0.724% vs. 5.302 ± 1.217%, 4.845 ± 1.859%, 4.900 ± 1.150%), Th2 (0.830 ± 0.151% vs. 0.588 ± 0.098%, 0.693 ± 0.128%, 0.637 ± 0.083%), T follicular helper (Tfh) (33.380 ± 4.715% vs. 21.700 ± 1.895%, 28.250 ± 5.038%, 26.720 ± 4.014%) and regulatory T (Treg) cells (4.252 ± 0.815% vs. 7.448 ± 1.439%, 6.089 ± 0.957%, 6.211 ± 0.880%) as assessed after 3 days of culture among all groups (Fig. 2C–F, Figs. S3, S4, S5, S6, Tables S3, S4, S5, S6).

### hUCMSC-EVs had no effect on CD19^+^ B cells in MRL/lpr mice splenic mononuclear cells in vitro

**Figure 3:**
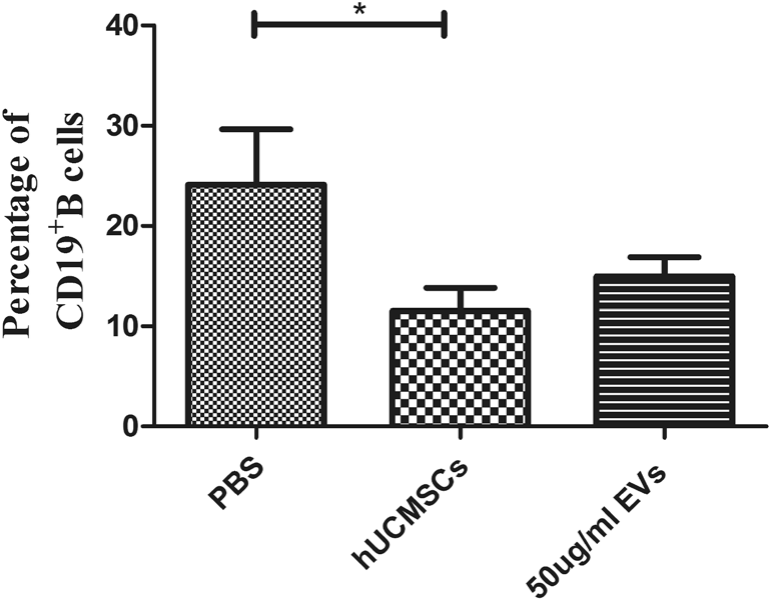
In vitro immunomodulatory effects of hUCMSCs and hUCMSC-EVs on MRL/lpr mouse splenic B cells. Flow cytometric analysis of the proportion of CD19^+^ B cell in splenic mononuclear cells. Data were expressed as the means ± SEM, and were analysed by Kruskal–Wallis test with Dunn's post hoc test, *P < 0.05.

In our previous experiment, we found that the dose of 25 μg/ml hUCMSC-EVs might be insufficient. The dose of 50 μg/ml hUCMSC-EVs cocultured with mouse splenic mononuclear cells activated by ODN 1826 for 3 days. The results showed that hUCMSCs (11.490 ± 2.291%) decreased CD19^+^ B cells compared with PBS (24.130 ± 5.518%) (*P* < 0.05), however, hUCMSC-EVs (14.930 ± 1.928%) have nonsignificant effect on CD19^+^ B cells (Fig. 3, Fig. S7, Table S7).

## The effects of hUCMSC-EVs on cytokines in co-culture supernatant

### hUCMSC-EVs had no effect on the cytokine of IFN-γ in supernatant in vitro

**Figure 4:**
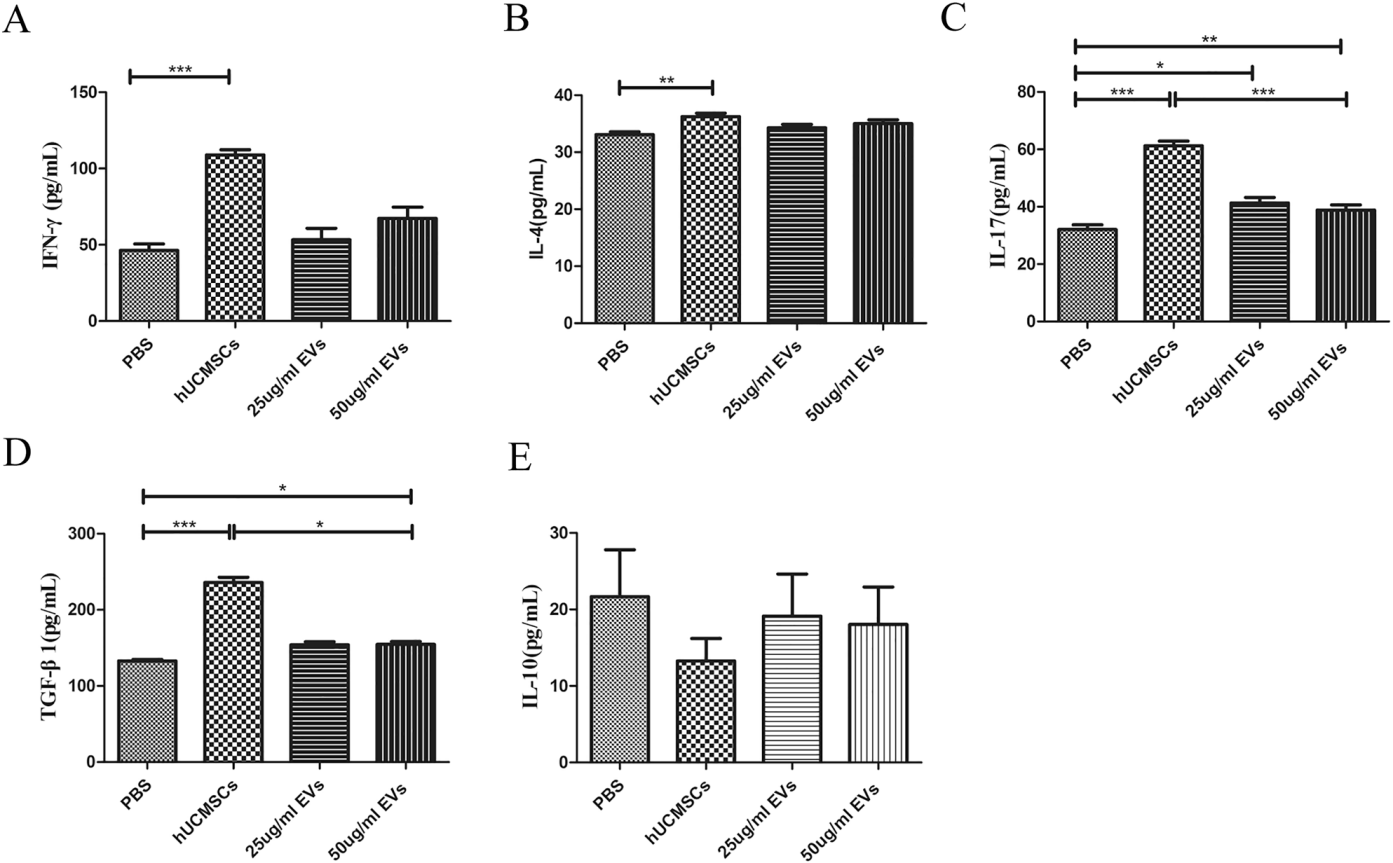
In vitro immunomodulatory effects of hUCMSCs and hUCMSC-EVs on cytokines (**A**–**E**). Culture media supernate levels of IFN-γ, IL-4, IL-17, TGF-β1 and IL-10 in each group. Data were expressed as the means ± SEM. Data were analysed by one-way ANOVA with Tukey's post hoc test or Kruskal–Wallis test with Dunn's post hoc test, *P < 0.05, **P < 0.01, ***P < 0.0001.

To understand the underlying mechanism of hUCMSC-EVs in modulating the immune response, we next examined the effects of hUCMSC-EVs on cytokine. Compared with PBS control group (46.340 ± 4.216%), hUCMSCs (108.900 ± 3.450%) treatment increase the concentration of IFN-γ in culture medium, while no difference was observed with hUCMSC-EVs coculture (53.310 ± 7.449% or 67.300 ± 7.421%) (Fig. 4A, Table S8).

### hUCMSC-EVs had no effect on the cytokine of IL-4 in supernatant in vitro

As shown, the level of IL-4 of the hUCMSCs group (36.230 ± 0.615%) was increased when compared with that of the PBS control group (33.100 ± 0.489%). Conversely, there was no significant difference between the hUCMSC-EVs groups (34.280 ± 0.593% or 35.020 ± 0.660%) and control groups (Fig. 4B, Table S9).

### hUCMSC-EVs increased the cytokine concentration of IL-17 in the supernatant in vitro

The results showed that the level of IL-17 was increased (*P* < 0.05) in the supernatant following 3 days incubation of the hUCMSCs (61.260 ± 1.611%) or hUCMSC-EVs (38.820 ± 1.798% or 41.290 ± 1.968%) compared with incubation of PBS (32.030 ± 1.675%) (Fig. 4C, Table S10). It indicates that hUCMSCs at least partially regulated the expression of IL-17 through hUCMSC-EVs.

### hUCMSC-EVs increased the cytokine concentration of TGF-β1 in the supernatant in vitro

As shown, hUCMSCs (235.800 ± 7.115%) or hUCMSC-EVs (154.200 ± 3.906% or 154.600 ± 3.772%) treatment increased the concentration of TGF-β in culture medium (Fig. 4D, Table S11) when compared with PBS treatment (132.800 ± 2.105%). It indicates that hUCMSCs at least partially regulated the expression of cytokine TGF-β1 through hUCMSC-EVs.

### hUCMSC-EVs had no effect on the cytokine of IL-10 in supernatant in vitro

Compared with PBS treatment (21.660 ± 6.140%), no difference of the level of IL-10 was detected in conditioned medium of splenic mononuclear cells treated with hUCMSCs (13.250 ± 2.966%) or hUCMSC-EVs (19.140 ± 5.488% or 18.050 ± 4.892%) (Fig. 4E, Table S12).

## Discussion

MSCs induce peripheral immunotolerance and regulate immune response, and the specific mechanism has not been fully elucidated. Limitation of heterogenicity in tissue-derived MSC-EVs as a drug MSC-EVs derived from different donors make it hard to keep quality consistency. A rigorous quality control system for MSC-EVs production is critical to reducing batch to batch variation. To tackle these challenges, MSC-EVs can also be derived from the same parental pluripotent stem cells (PSCs) derived-MSCs^26^. Most recently, GMP-grade MSCs derived from hiPSCs have been used in refractory GVHD in clinical trials^27^. EVs produced from PSC-MSCs may provide an alternative cellular source that overcomes many limitations of heterogenicity of MSC-EVs^28^. EVs are one of the important paracrine cargoes secreted by almost all cell types. Current researches suggest that MSC-EVs are a promising candidate for a new cell-free therapy of a wide range of immune diseases. To date, among a series of published researches on the animal models or clinical trials of SLE, there is no experimental study reporting the immunoregulatory effects of MSC-EVs on T or B cells. In this study, we reported that both hUCMSCs and hUCMSC-EVs inhibited CD4^+^ T cells and increase the proportion of Th17 cells in splenic mononuclear cells; hUCMSCs inhibited B cells; both hUCMSCs and hUCMSC-EVs had no significant effects on Th1, Th2, Tfh and Treg cells. Meanwhile, both hUCMSCs and hUCMSC-EVs promote the expression of IL-17 and TGF-β1; hUCMSCs promote the production of IFN-γ and IL-4; hUCMSCs and hUCMSC-EVs had no significant regulatory effect on cytokine IL-10. We report that hUCMSC-EVs at least partly mimic the immunomodulatory effects of MSCs in SLE, such as inhibiting CD4^+^ T cells, promoting Th17 cells, and increasing the production of IL-17 and TGF-β1. Therefore, hUCMSC-EVs play novel and potential immunoregulation roles in SLE.

SLE is a chronic systemic inflammatory autoimmune disease characterized by the breakdown of autoimmune tolerance. Previous studies have shown that abnormal CD4^+^ T cell apoptosis or proliferation may play a key role in the initiation and promotion of autoreactive humoral immunity. The gene mutation of lymphoproliferation (Lpr) in MRL/lpr mice resulted in a lack of functional *Fas* receptor in vivo^29,30^, which mainly promoted T cell expansion^29^ and hindered T cell apoptosis^30^. On the one hand, renal infiltration of CD4^+^ T cells promoted the development of Lupus nephritis in MRL/lpr mice^31^. On the other hand, CD4^+^ T cell-deficient MRL/lpr mice produced significantly less autoantibody and prolonged survival^32^. Furthermore, anti-CD4 monoclonal antibody therapy for SLE significantly reduced the incidence of vasculitis, and glomerulonephritis, and significantly lowered the levels of antinuclear antibody, total IgG, and anti-dsDNA^33^. Studies showed that MSCs delayed disease progression by promoting CD4^+^ T cell apoptosis or inhibiting CD4^+^ T cell proliferation in MRL/lpr mice^1,34^. In this study, hUCMSCs or hUCMSC-EVs inhibited CD4^+^ T cells in MRL/lpr mice. Therefore, hUCMSCs may slow the progression of lupus disease by inhibiting CD4^+^ T cells through paracrine extracellular vesicles.

IFN-γ and IL-4 are two typical cytokines representing the Th1 and Th2 subsets, respectively. Th2 cells are mainly involved in phenotypic changes from primary T cells to effector T cells and humoral immune activation^31,32^. Clinically, SLE is diagnosed by detecting a variety of pathogenic autoreactive antibodies against nucleoproteins and nucleic acids, hence Th2 cells may play an important role in the pathogenesis of SLE. Previous studies have differed on the therapeutic effect of MSCs on lupus disease, For example, MSCs promote Th1 cell differentiation^1,2,35^ and Th2 cell differentiation^36^ or inhibit immune response of Th1 and Th2 in lupus models or patients^37^. The results in this study indicated that hUCMSCs or hUCMSC-EVs have no effects on Th1 and Th2 cells in MRL/lpr mice in vitro, while hUCMSCs increased the production of cytokines IFN-γ and IL-4 in the supernatant. This phenomenon explained by the following reasons: (1) the cytokine concentration in the supernatant measured by ELISA was actually the total amount of cytokines produced by mononuclear cells. Cell types determined by flow cytometry for intracellular cytokine detection were actually to confirm whether mononuclear cells differentiate into Th1 or Th2 cells at the single-cell level. (2) The cytokine network of SLE is extremely complex, which is related to the disease state, the specific tissues involved, and the genetic constitution. These diseases present complex symptoms, suggesting that the cytokine network in SLE may also be very complicated. These factors may contribute to the differences in results of various laboratories.

There were researches indicating that MSCs promoted the production of Th17 cells and Treg cells in peripheral blood mononuclear cells, as well as production of IL-17 and TGF-β in SLE patients^38^. In non-SLE diseases, MSCs also promoted the differentiation of activated CD4^+^ T cells into Th17 cells and the production of IL-17. Our study indicated that hUCMSCs or hUCMSC-EVs promoted the differentiation of CD4^+^ T cells into Th17 cells and increased the levels of IL-17 and TGF-β1.

Follicular helper T cells (Tfh) as another subset of CD4^+^ T cells, also play an important role in B cell differentiation, maturation and antibody secretion^39^. These results showed that hUCMSCs inhibited Tfh cells, while hUCMSC-EVs only had a tendency to inhibit Tfh cells. The research demonstrated that MSCs inhibit the differentiation of CD4^+^ T cells into Tfh cells through cell-to-cell contact^40–42^. Whether hUCMSCs can inhibit Tfh cell differentiation through paracrine extracellular vesicles needs to be further verified by expanding the sample size or increasing the intervention amount of EVs.

IL-10 is a multifunctional cytokine that plays an important role in regulating the growth and differentiation of B cells and the production of autoantibodies^43^. Most IL-10 came from monocytes and B cells, with a small amount from T cells^44,45^. In 1993, it was first reported that PBMCs in newly diagnosed SLE patients produced more IL-10 than healthy control group^46^, and serum IL-10 was significantly associated with lupus disease activity and anti-ds-DNA titers^47^. Anti-IL-10 monoclonal antibody therapy can reduce urinary protein and autoreactive IgG levels and inhibit in vitro cellular immune responses of peripheral blood mononuclear cells in SLE patients^48^. IL-10 can also inhibit the production of active TGF-β^49^. Hence IL-10 may be the hub connecting Treg cells, Tfh cells and B cells. Previous studies have also shown that MSCs alleviate lupus-like diseases by reducing the number of CD19^+^ B cells^50^. The secretions of MSCs reduce serum IL-10 levels in lupus mice^51^. This study demonstrated that hUCMSCs inhibited B cells; hUCMSC-EVs had no effects on B cells, and hUCMSCs or hUCMSC-EVs had no significant regulatory effects on IL-10. It is possible that hUCMSCs regulate B cells through other mechanisms, or the small sample size or intervention amount of EVs in this study resulted in no statistical significance among groups.

In summary, hUCMSCs exert immunoregulatory effects on SLE at least partially through hUCMSC-EVs in vitro, and hUCMSC-EVs play a novel and potential regulator roles in SLE. However, we lack in vivo experiments to further verify the regulatory effects of hUCMSC-EVs on immune cells in MRL/lpr mice and the therapeutic effect on the condition of mice.

## Conclusion

We found that hUCMSCs and hUCMSC-EVs suppressed the total CD4^+^ T cells, regulate the proportion of Th17 cells and promote the expression of soluble cytokines IL-17 or TGF-β1 in vitro. These data suggested that hUCMSC-EVs are promising in regulating the immune cells and cytokines in the similar way as treatment with hUCMSCs in SLE. This study may provide a theoretical basis for the application of MSC-EVs in the treatment of autoimmune diseases.

## Methods

### Experimental animal

Female MRL/lpr mice (stock number 000485 and induced from Jackson laboratory, USA) were purchased from Shanghai SLAC Laboratory Animal Co. Ltd. and raised under the specific-pathogen-free facilities in the Laboratory Animal Center of the Second Xiangya Hospital, Central South University until they were used. All experimental applications were carried out in accordance with ARRIVE guidelines. The MRL/lpr mouse used in this experiment is a classic animal model of SLE. The onset of autoimmune diseases in MRL/lpr mice was monitored by measuring proteinuria (100 mg/dl), and MRL/lpr mice have symptoms such as lymphoproliferation and skin damage, which are in line with the description in Jackson Laboratory. Please see the Jackson laboratory website for details: https://www.jax.org/jax-mice-and-services. Female MRL/lpr mice were euthanized at age of 20 weeks and mononuclear cells were isolated from the spleen for in vitro experiments. Splenic mononuclear cells from the same mouse were separated into four groups (2 × 10^6^cell/group) as phosphate-buffered saline (PBS) control group, MSC group, 25 µg/ml hUCMSC-EVs group, 50 µg/ml hUCMSC-EVs group. The Ethical approval was obtained from the Institutional Animal Care and Use Committee of the Second Xiangya Hospital, Central South University (Approval No. 2020344) for the study, animal experimentation guidelines were exactly followed.

### hUCMSC-EVs extraction and identification

The hUCMSCs were subcultured and expanded by the primary hUCMSCs isolated from healthy human umbilical cord Wharton’s jelly. The 3–8 passages of hUCMSCs were cultured in serum-free complete medium (Clin-Biotechnology, China). The hUCMSCs reached 80% confluency, the MSCs serum-free complete medium was removed and the surface of the cells was cleaned with PBS for twice, followed by cultured in MSC basic medium (Clin-Biotechnology, China) for 3 days. Sequentially for 15 min at 300*g*, 20 min at 2000*g*, and 30 min at 10,000*g*. After each centrifugation step, the pellet was removed and discarded. The centrifuged supernatant was passed through a 0.22 µm filter and then transferred to Ultra-15 10-KDA MWCO test tubes (Merck Millipore, Ireland), followed by centrifugating at 4000*g* for 30 min to concentrate supernatant. Finally, The centrifugation was transferred to ultracentrifuge tubes and centrifuged using an ultracentrifuge (SW 41 Ti 41,000 RPM, Beckman Optima XPN-100, USA) for 2 h at 120,000*g*. The pellet was resuspended in PBS. All centrifugation processes were carried out under 4 °C. The total protein concentration of EVs was measured by BCA protein quantitation method (Thermo Scientific, USA).

Ten microliter EVs were dropped onto the ultrathin carbon film copper mesh, then EVs were treated with glutaraldehyde, uranium oxalate, and methylcellulose-UA in turn, and after drying, the morphology of EVs was evaluated by the transmission electron microscope (TEM) (Hitachi, Japan). The purified EVs were diluted 10,000 times, and Nanoparticle Tracking Analysis (NTA) (ZetaView, Germany) was used to measure the particle size distribution of EVs. To detect EVs specific markers and negative markers, the samples were incubated with the antibodies of anti-CD9, anti-TSG101, and anti-Calnexin (Abcam, UK), and then incubated with enzyme-labeled anti-rabbit IgG antibody (Sigma, USA), and finally a western blot developer machine was used.

### Mononuclear cells were co-cultured with hUCMSCs or hUCMSC-EVs

Spleen mononuclear cells were isolated by density gradient centrifugation with mouse Ficoll according to the manufacturer's instructions. Briefly, female MRL/lpr mice for 20 weeks were euthanized and the spleen was removed aseptically. Spleen grinding liquid were layered over Ficoll-Paque PREMIUM 1.084 g/ml (GE, catalog No. 17544602-1, USA) and centrifuged at 400*g* for 30–40 min at 18–20 °C. The mononuclear cells were collected at the interface, then the cells were mixed with 3 times the volume of PBS to wash twice by centrifugation at 60–100 g for 10 min at 18–20 °C. Then the cells were resuspended in RPMI1640 (Gibco, USA) supplemented with 10% fatal bovine serum (Gibco, USA). The final concentration of mononuclear cells was adjusted to 2 × 10^6^/ml. hUCMSCs were pretreated with 10 µg/ml mitomycin C (Sigma, USA) and inoculated at a density of 2 × 10^5^/well into a 12-well plate (Corning, USA) and added with RPMI1640 medium (Gibco, USA) containing 10% fetal bovine serum, 100 U/ml penicillin as well as 100 mg/ml streptomycin overnight. On the second day, 1 ml suspension of 2 × 10^6^/ml mouse splenic mononuclear cells was added into the well with hUCMSCs, and the ratio of hUCMSCs and splenic mononuclear cells = 1:10. In the experimental well of hUCMSC-EVs immune response to T cells, to activate T cells under Th0 condition, 1.0 µg/ml anti-CD3e and anti-CD28 were added to the co-culture mentioned above. In order to activate B cells, 6 µg/ml ODN 1826 B cell activators were added to the above co-culture system. Then the final volume of each well was adjusted to 1.2 ml with PBS and hUCMSCs were co-cultured with mononuclear cells for 3 days. In the hUCMSC-EVs group, fresh hUCMSC-EVs preparations of 25 µg/ml or 50 µg/ml were added to study the effect of hUCMSC-EVs in the same procedure and time. As 25 µg/ml hUCMSC-EVs had no significant regulation effect on T cells, only 50 µg/ml hUCMSC-EVs were used to study the regulation effect of hUCMSC-EVs on B cells. The control group was treated with PBS.

### CD4^+^ T cell subsets and CD19^+^ B cells were detected by a flow cytometer

Suspended mononuclear cells were recovered from the culture medium, washed with PBS, and the proportions of CD4^+^ T cell subsets and CD19^+^ B cells were detected by a flow cytometer (Cytek, USA). Cells were stained with anti-mouse antibodies that are fluorescent-conjugated for CD4 (BD Pharmingen, USA), IFN-γ (BD Pharmingen, USA), IL-4 (BD Pharmingen, USA), IL-17a (BD Pharmingen, USA), CD25 (BD Pharmingen, USA), Foxp3 (BD Pharmingen, USA), CD185 (BD Pharmingen, USA), CD19 (BD Pharmingen, USA). CD4^+^IFN-γ^+^ T cells, CD4^+^IL-4^+^ T cells, CD4^+^IL-17^+^ T cells, CD4^+^CD25^+^Foxp3^+^ T cells and CD4^+^CD185^+^ T cells were thought as Th1, Th2, Th17, Treg and Tfh cells, respectively. For intracellular staining, the cells were treated with cytokine stimulation blockers (BD Pharmingen, USA). The Flowjo10.5.3 program was used to analyze the data.

### Cytokines were measured by ELISA

Cytokine levels in the co-culture supernatant were measured by commercial ELISA kits. Mice were tested for IFN-γ (USCN, catalog No. SEA049Mu, China), IL-4 (Elabscience, catalog No. E-EL-M0043c, China), IL-17a (Elabscience, catalog No. E-EL-M0043c, China), TGF-β1 (USCN, catalog No. SEA124Mu, China), and IL-10 (USCN, catalog No. SEA056Mu, China) in accordance with the manufacturer's instructions.

### Statistical analysis

Comparisons between groups were performed with one-way analysis of variance (ANOVA) with Tukey's multiple comparison test for data normal distribution for all groups except for except for the data, which was compared with Kruskal–Wallis test with Dunn's multiple comparison test for data non-normal distribution. We performed statistical analyses and maps with GraphPad Prism 5 software and considered a *P* value less than 0.05 as significant. Data are shown as means ± standard error of mean (means ± SEM). In the results, the explanation of the descriptive statistics used mean and standard deviation.

### Ethics approval and consent to participate

The studies involving animals were reviewed and approved by the institutional ethics board of the Second Xiangya Hospital of Central South University (No. 2020344).

## Supplementary Information

Supplementary Figures.

Supplementary Tables.

## Data Availability

The original contributions presented in the study are included in the article/supplementary material. Further inquiries can be directed to the corresponding author.

## Abbreviations

hUCMSCs: Human umbilical cord mesenchymal stem cells
hUCMSC-EVs: Human umbilical cord mesenchymal stem cells derived extracellular vesicles
PBS: Phosphate-buffered saline
IFN-γ: Interferon-γ
IL: Interleukin
TGF-β1: Transforming growth factor-β1
Th: T helper
Tfh: T follicular helper
Treg: Regulatory T
SLE: Systemic lupus erythematosus
HSCs: Hematopoietic stem cells
GVHD: Graft-versus-host disease
LN: Lupus nephritis
NTA: Nanoparticle tracking analysis
