# Supplementary material for: Human umbilical cord mesenchymal stem cells derived extracellular vesicles regulate acquired immune response of lupus mouse in vitro
Source: Sci Rep. 2022 Jul 30;12:13101. doi: 10.1038/s41598-022-17331-8 (PMC9338971; doi:10.1038/s41598-022-17331-8)
Supplement: Supplementary file 1 — Supplementary Figures. [file 41598_2022_17331_MOESM1_ESM.pdf]

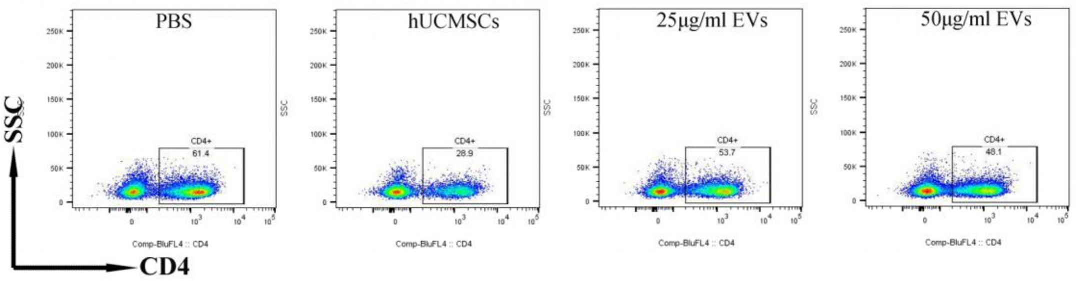

Figure S1. Flow cytometric analysis shows the proportion of CD4^{+}T cells in spleen mononuclear cells of MRL/lpr mice in each group.

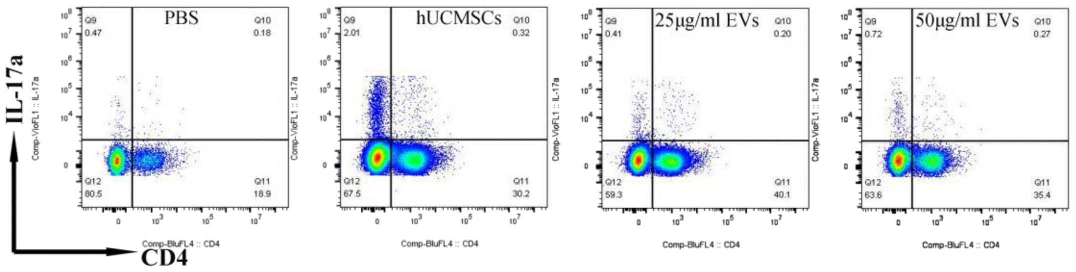

Figure S2. Flow cytometric analysis shows the proportion of Th17 cells in spleen mononuclear cells of MRL/lpr mice in each group.

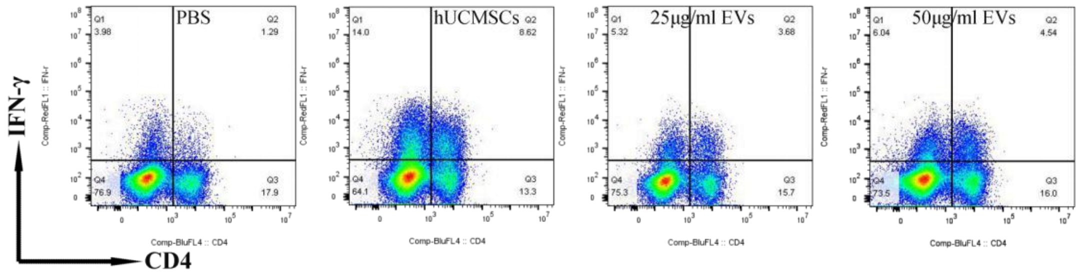

Figure S3. Flow cytometric analysis shows the proportion of Th1 cells in spleen mononuclear cells of MRL/lpr mice in each group.

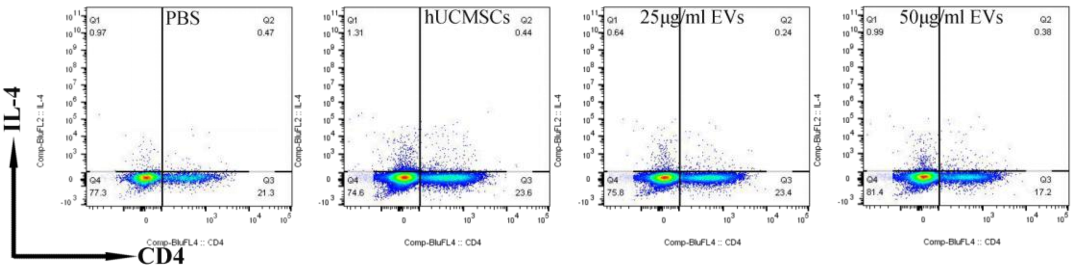

Figure S4. Flow cytometric analysis shows the proportion of Th2 cells in spleen mononuclear cells of MRL/lpr mice in each group.

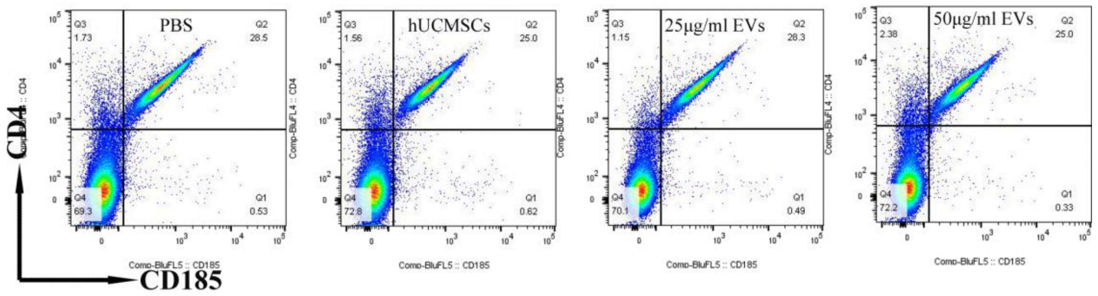

Figure S5. Flow cytometric analysis shows the proportion of Tfh cells in spleen mononuclear cells of MRL/lpr mice in each group.

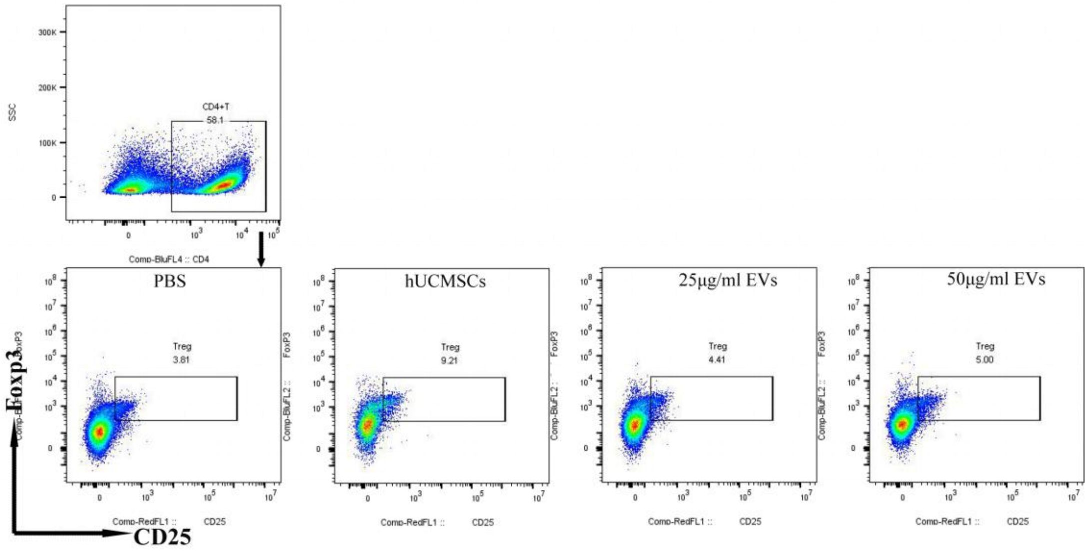

Figure S6. Flow cytometric analysis shows the proportion of Treg cells in spleen mononuclear cells of MRL/lpr mice in each gro

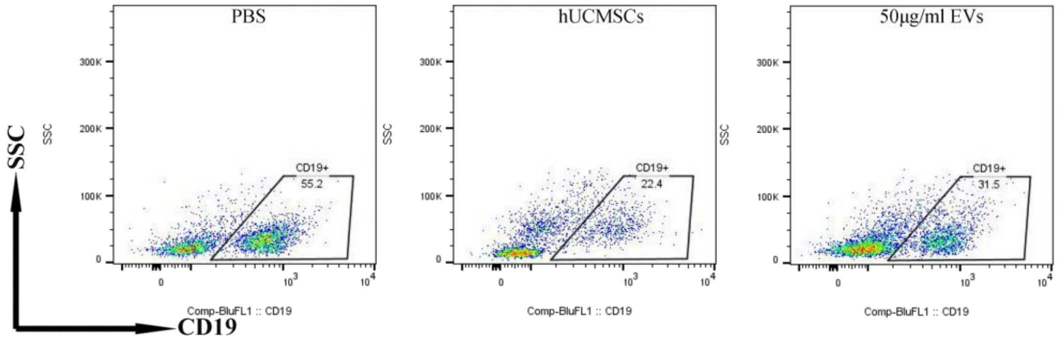

Figure S7. Flow cytometric analysis shows the proportion of CD19^{+}B cells in spleen mononuclear cells of MRL/lpr mice in each group.

A

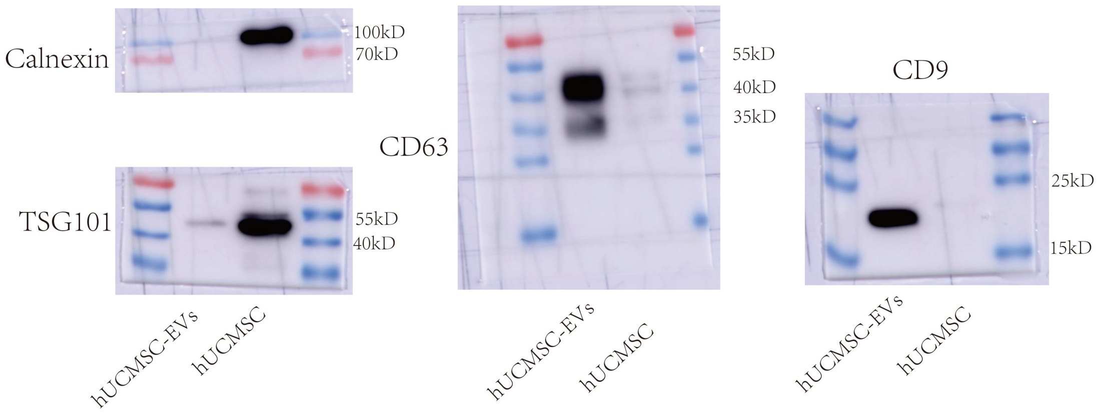

B

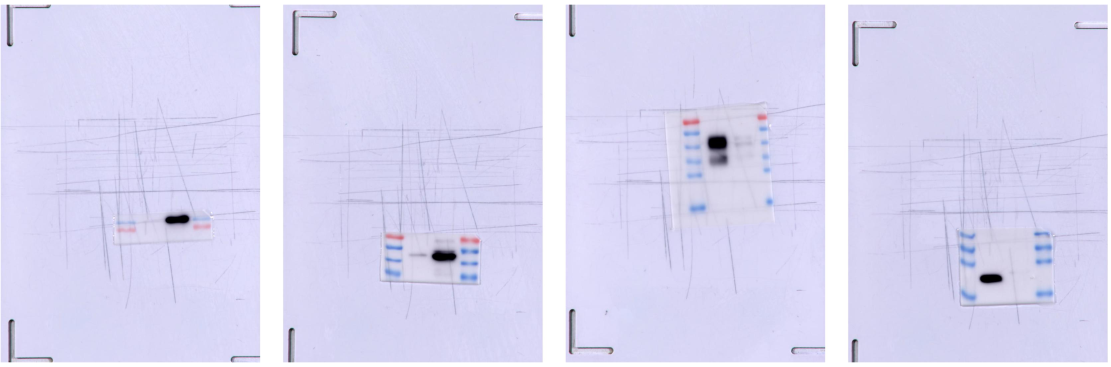

Figure S8. (A) EV markers of CD9, CD63 and TSG101 were positive in the hUCMSC-EVs.Calnexin was detected in hUCMSCs but not in hUCMSC-EVs.(B) The cropped images showed all blots. Before incubating with the primary antibody, that is, in the step of trarsmembrane, the gel was cropped that at least two markers adjacent to the target band were reserved according to the position of molecular size markers.
