# Supplementary material for: Human umbilical cord mesenchymal stem cells derived extracellular vesicles regulate acquired immune response of lupus mouse in vitro
Source: Sci Rep. 2022 Jul 30;12:13101. doi: 10.1038/s41598-022-17331-8 (PMC9338971; doi:10.1038/s41598-022-17331-8)
Supplement: Supplementary file 2 — Supplementary Tables. [file 41598_2022_17331_MOESM2_ESM.pdf]

Table S1. Tukey's multiple comparison test in one-way analysis of variance in CD4^{+}T cells

Tukey's Multiple Comparison Test	Mean Diff.	q	*P* < 0.05	Summary	95% CI of diff
PBS vs UCMSCs	9.534	4.175	Yes	*	1.115 to 17.95
PBS vs 25ug/ml EVs	4.862	2.129	No	ns	-3.558 to 13.28
PBS vs 50ug/ml EVs	8.633	3.780	Yes	*	0.2137 to 17.05
UCMSCs vs 25ug/ml EVs	-4.672	2.081	No	ns	-12.95 to 3.606
UCMSCs vs 50ug/ml EVs	-0.9010	0.4012	No	ns	-9.179 to 7.377
25ug/ml EVs vs 50ug/ml EVs	3.771	1.680	No	ns	-4.507 to 12.05

Table S2. Dunn's multiple comparison test in Kruskal-Wallis test in Th17 cells

Dunn's Multiple Comparison Test	Difference in rank sum	*P* < 0.05	Summary
PBS vs hUCMSCs	-10.25	Yes	*
PBS vs 25μg/ml EVs	-3.500	No	ns
PBS vs 50μg/ml EVs	-8.975	Yes	*
hUCMSCs vs 25μg/ml EVs	6.750	No	ns
hUCMSCs vs 50μg/ml EVs	1.275	No	ns
25μg/ml EVs vs 50μg/ml EVs	-5.475	No	ns

Table S3. Kruskal-Wallis test of Th1 cells in all groups

Kruskal-Wallis test	
P value	0.0767
Exact or approximate *P* value?	Gaussian Approximation
P value summary	ns
Do the medians vary signif. (*P* < 0.05)	No
Number of groups	4
Kruskal-Wallis statistic	6.853

Tale S4. Kruskal-Wallis test of Th2 cells in all groups

Kruskal-Wallis test	
P value	0.6452
Exact or approximate *P* value?	Gaussian Approximation
P value summary	ns
Do the medians vary signif. (*P* < 0.05)	No
Number of groups	4
Kruskal-Wallis statistic	1.663

Tale S5. Kruskal-Wallis test of Tfh cells in all groups

Kruskal-Wallis test	
P value	0.2004
Exact or approximate *P* value?	Gaussian Approximation
P value summary	ns
Do the medians vary signif. ($P < 0.05$)	No
Number of groups	4
Kruskal-Wallis statistic	4.637

Table S6. One-way analysis of variance of Treg cells in all groups

One-way analysis of variance	
P value	0.2218
P value summary	ns
Are means signif. different? ($P < 0.05$)	No
Number of groups	4
F	1.527
R squared	0.1005

Tale S7. Dunn's multiple comparison test in Kruskal-Wallis test in $CD19^{+}$B cells

Dunn's Multiple Comparison Test	Difference in rank sum	$P < 0.05$	Summary
PBS vs hUCMSCs	10.25	Yes	*
PBS vs 50μg/ml EVs	4.432	No	ns
hUCMSCs vs 50μg/ml EVs	-5.818	No	ns

Tale S8. Dunn's multiple comparison test in Kruskal-Wallis test of IFN-γ

Dunn's Multiple Comparison Test	Difference in rank sum	$P < 0.05$	Summary
PBS vs hUCMSCs	-22.85	Yes	***
PBS vs 25μg/ml EVs	-2.700	No	ns
PBS vs 50μg/ml EVs	-8.450	No	ns
hUCMSCs vs 25μg/ml EVs	20.15	Yes	***
hUCMSCs vs 50μg/ml EVs	14.40	Yes	*
25μg/ml EVs vs 50μg/ml EVs	-5.750	no	ns

Tale S9. Dunn's multiple comparison test in Kruskal-Wallis test of IL-4

Dunn's Multiple Comparison Test	Difference in rank sum	*P* < 0.05	Summary
PBS vs hUCMSCs	-18.00	Yes	**
PBS vs 25μg/ml EVs	-6.800	No	ns
PBS vs 50μg/ml EVs	-10.60	No	ns
hUCMSCs vs 25μg/ml EVs	11.20	No	ns
hUCMSCs vs 50μg/ml EVs	7.400	No	ns
25μg/ml EVs vs 50μg/ml EVs	-3.800	No	ns

Table S10. Tukey's multiple comparison test in one-way analysis of variance in IL-17

Tukey's Multiple Comparison Test	Mean Diff.	q	*P* < 0.05	Summary	95% CI of diff
PBS vs UCMSCs	-29.24	16.54	Yes	***	-35.98 to -22.50
PBS vs 25ug/ml EVs	-6.794	3.842	Yes	*	-13.54 to -0.05284
PBS vs 50ug/ml EVs	-9.258	5.236	Yes	**	-16.00 to -2.516
UCMSCs vs 25ug/ml EVs	22.44	12.69	Yes	***	15.70 to 29.18
UCMSCs vs 50ug/ml EVs	19.98	11.30	Yes	***	13.24 to 26.72
25ug/ml EVs vs 50ug/ml EVs	-2.464	1.393	No	ns	-9.205 to 4.278

Tale S11. Dunn's multiple comparison test in Kruskal-Wallis test of TGF-β1

Dunn's Multiple Comparison Test	Difference in rank sum	P < 0.05	Summary
PBS vs hUCMSCs	-29.15	Yes	***
PBS vs 25μg/ml EVs	-13.20	No	ns
PBS vs 50μg/ml EVs	-14.25	Yes	*
hUCMSCs vs 25μg/ml EVs	15.95	Yes	*
hUCMSCs vs 50μg/ml EVs	14.90	Yes	*
25μg/ml EVs vs 50μg/ml EVs	-1.050	No	ns

Tale S12. Kruskal-Wallis test of IL-10 in supernatant in all groups

Kruskal-Wallis test	
P value	0.3498
Exact or approximate *P* value?	Gaussian Approximation
P value summary	ns
Do the medians vary signif. (*P* < 0.05)	No
Number of groups	4
Kruskal-Wallis statistic	3.284
